# Modelling the impact of different intervention packages for malaria control under varying intensities of pyrethroid resistance

**DOI:** 10.1186/s12936-025-05633-x

**Published:** 2025-11-19

**Authors:** Hamenyimana E. Gervas, Maranya M. Mayengo, Yeromin P. Mlacha, Halfan S. Ngowo, Fredros O. Okumu, Prashanth Selvaraj

**Affiliations:** 1 Nelson Mandela African Institution of Science and Technology (NM-AIST), Arusha, Tanzania; 2 Environmental Health and Ecological Science Department, Ifakara Health Institute (IHI), Ifakara, Tanzania; 3 Department of Mathematics and Statistics, University of Dodoma (UDOM), Dodoma, Tanzania; 4 Institute for Disease Modeling, Gates Foundation, Seattle, USA; 5 University of Glasgow, Scotland, UK

**Keywords:** Insecticide resistance, *Anopheles funestus*, *Anopheles arabiensis*, Resistance intensity, Mathematical modelling, Integrated vector control

## Abstract

**Background:**

Malaria control in sub-Saharan Africa faces significant challenges from biological threats, such as insecticide resistance and adaptive vector behaviours, as well as increasing financial constraints, which necessitate strategic intervention planning to maximize impact. This study assesses the effectiveness of combining vector control methods, case management, and immunoprevention to reduce malaria in Tanzania, considering varying intensities of insecticide resistance in the main vector species.

**Methods:**

A compartmental model was developed to simulate malaria transmission, incorporating the dominant vectors: *Anopheles funestus* (anthropophilic and endophilic) and *Anopheles arabiensis* (zoophilic and exophilic). The model was used to analyse the impacts of insecticide-treated nets (ITNs), indoor residual spraying (IRS), and biolarvicides, used singly or in combinations, under varying intensities of pyrethroid resistance. The analysis was further expanded to explore the impacts of adding case management (treatment using artemisinin-based combinations) and immunization (RTS,S/AS01 and R21/Matrix-M vaccines).

**Results:**

At moderate levels of pyrethroid resistance (50%), achieving at least 71% ITN coverage combined with either 50% IRS or 32% biolarvicide coverage reduces the effective reproduction number ($R_e$) to below 1. However, at high resistance levels (exceeding 75%), the effective reproduction number ($R_e$) consistently remains above 1, irrespective of the type or combination of vector control interventions. Adding immunization ($\ge$ 40% coverage) to ITNs (80% coverage), along with effective treatment (80% coverage), can further reduce the proportion of infectious individuals to <20% and $R_e$ below 1, even under high resistance intensities.

**Conclusions:**

Compared to ITNs alone, combining ITNs with IRS and/or biolarvicides greatly improves malaria control at low to moderate intensities of pyrethroid resistance but yields no additional benefits at high resistance intensities. However, integrating these vector control strategies with immunization and effective case management using artemisinin-based combination therapy (ACT) further enhances impact by reducing both parasite transmission and the infectious reservoir.

## Background

Since 2000, malaria control interventions have prevented about 2.2 billion cases and 12.7 million deaths [1], nearly 70% of these gains being attributed to the scale up of key vector control approaches, mainly insecticide-treated nets (ITNs) and indoor residual spraying (IRS) [2, 3]. However, this success has been plateauing and there is evidence of malaria cases surging in a number of sub-Saharan African countries in recent years [4]. Several reasons contribute to this rise. Besides weak health systems and poor living conditions [5], there is a surge in biological threats, particularly the widespread resistance to drugs [6], and commonly used insecticides [3, 7]. Other challenges include the outdoor biting behaviour of mosquitoes [8, 9], human behaviour and activities that limit the efficacy of ITNs [10] and climate change [4]. These factors are directly and indirectly impeding progress towards the WHO’s Global Technical Strategy (GTS) target of a 90% reduction in malaria burden by 2030 [11].

Insecticide resistance, particularly to pyrethroids, poses a serious threat to malaria vector control programs and has been reported in over 75% of malaria-endemic countries [3, 12]. The concurrent use of pyrethroids in both agriculture and public health significantly contributes to the emergence and spread of insecticide resistance, as over 90% of agricultural chemicals are also used in vector control [13, 14]. In response to pyrethroid resistance, novel formulations and combinations of insecticides are being developed and advocated by partners [12, 15]. Among these innovations are next-generation long-lasting insecticidal nets (LLINs) that combine a pyrethroid with a second active component [12]. In some nets, the additional component is the synergist piperonyl butoxide (PBO), as in pyrethroid-PBO nets like Olyset$^\circledR$ Plus and PermaNet$^\circledR$ 3.0 ITNs (pyrethroid-PBO ITNs) [16], while in others it is the insecticide chlorfenapyr and pyriproxyfen, as exemplified by the dual-active Interceptor$^\circledR$ G2 (IG2) and pyrethroid-pyriproxyfen ITNs [12, 15], and PRONet$^\circledR$ [17]. The impact of these new ITN types have been strongly supported by studies targeting pyrethroid-resistant mosquitoes [18, 19]. Additionally, IRS, unlike ITNs, can use multiple insecticide classes beyond pyrethroids and is adopted by some countries, particularly in high-transmission settings [20]. IRS can also be part of resistance management plans, though it faces challenges like high logistical costs and difficulties in managing the insecticide life cycle [21].

In Tanzania, pyrethroid resistance is reported in over $80\%$ of the vector surveillance sentinel sites, with resistance confirmed in all primary malaria vectors: *Anopheles gambiae sensu stricto (s.s.)*, *Anopheles arabiensis*, and *Anopheles funestus* [22, 23]. To combat resistance, the National Malaria Control Programme (NMCP) is deploying pyrethroid-PBO ITNs in pyrethroid-resistant regions and has previously implemented IRS using organophosphate or neonicotinoid insecticides [24]. However, following the discontinuation of IRS in 2021 [25], the NMCP is considering the new forms of ITNs in areas previously covered by IRS and standard ITNs, while mobilizing alternative resources to support the potential re-introduction of IRS [26]. Additionally, the integration of biolarvicides (such as Bacillus thuringiensis israelensis (Bti) and Bacillus sphaericus (Bs)) in conjunction with ITNs has been suggested as an additional strategy to mitigate the emergence and spread of insecticide resistance in most regions [26]; and is already piloted in one region in North-eastern Tanzania [27, 28].

Going forward, comprehensive studies, are crucial to clarify the relationships between insecticide resistance and malaria epidemiology, and understand the impact of the current and proposed strategies, including combinations of vector control tools and other interventions [29]. Mathematical models are valuable tools for shaping public health strategies, particularly in disease control, policy formulation, and decision-making. They help address complex, costly, and hard-to access issues, even in contexts with limited field data [30, 31]. Since Sir Ronald Ross’s pioneering malaria transmission model [30], numerous models have analysed transmission dynamics, control measures, and insecticide resistance in mosquitoes [32–35]. Despite incorporating insecticide resistance intensity to assess the impact of supplementary tools alongside ITNs and IRS [32, 33, 36–44], most models do not account for the variation in resistance intensities and differences in the behaviour and responses of different vector species.

This study introduces a novel mathematical approach to simulate malaria transmission dynamics, accounting for varying insecticide resistance intensities in *An. arabiensis* and *An. funestus* (here referring to *An. funestus s.s.*) populations in Tanzania. The primary goal was to evaluate the impact of integrating different vector control tools on reducing malaria transmission, accounting for varying levels of insecticide resistance and the behaviours of the main vector species. Additionally, the model was expanded to evaluate the impacts of effective case management with first line drugs and immunization on reducing malaria burden.

## Methods

### Model development

A novel compartmental model is developed to describe the dynamics of malaria transmission, incorporating both susceptible and resistant sub-populations of *An. arabiensis* and *An. funestus*, as well as four sub-classes of the human population, as follows: at time $t \ge 0$, the human population is divided into: those who are susceptible, $S_H$ (healthy people but can contract an infection at any time), exposed, $E_H$ (infected people but cannot transmit disease), infectious, $I_H$ (infected people who can transmit disease), and recovered individuals, $R_H$ (malaria survivors). The recovered people have the partial immunity, so they can still be susceptible at rate, $\omega$. Humans die naturally at per capita rate, $\mu _H$ whereas those who are infected recover at a rate $\gamma$ and die at per capita rate, $\tau$. It is assumed that humans are recruited through new births and immigration at rate, $\Pi _H$. Thus, the total population of humans $(N_H)$ is given by

1 $$\begin{aligned} N_H=S_H+E_H+I_H+R_H. \end{aligned}$$

On the other hand, the *An. arabiensis* and *An. funestus* adult mosquito populations are divided into insecticide susceptible (non-resistant) (variables with the subscripts *A* and *B* respectively) and resistant sub-classes (variables with the subscripts *r* and *R* respectively). Then, $S_A$, $S_r$, $S_B$ and $S_R$ signify the mosquito classes that are susceptible to malaria parasites, and the parasite-exposed classes are denoted by $E_A$, $E_r$, $E_B$ and $E_R$. The infectious mosquitoes are represented by classes $I_A$, $I_r$, $I_B$ and $I_R$. The state variables $L_A$ and $L_B$ represent the immatures (larvae) of *An. arabiensis* and *An. funestus* mosquitoes respectively. Only the infectious stages of mosquitoes can transfer parasites to people, despite the fact that all exposed mosquitoes are infected (See Tables 1 and 2 for detailed descriptions of all variables and parameters).

In contrast to susceptible humans who become ill when bitten by infectious mosquitoes, the susceptible mosquitoes get infected when they bite infectious humans. It is also considered that, all newborn humans and mosquitoes are susceptible, and there is no direct transmission from one human to another or from one mosquito to another. The model also represents *An. arabiensis* and *An. funestus* mosquitoes as having different intensities of insecticide resistance, $e_1$ and $e_2$ respectively (resistance intensity, defined as strength of mosquito resistance to insecticides, characterized by the ability to survive exposure to increasing concentrations ($1 \times$, $5 \times$ and $10 \times$) of a standard insecticide [21, 45]). The mosquitoes are assumed to be recruited at $\Lambda _1$ and $\Lambda _2$ respectively. As a result, the total adult mosquito population $(N_v)$ is the sum of the total adult populations of *An. arabiensis* ($N_1$) and *An. funestus* ($N_2$), defined as

2 $$\begin{aligned} N_v = N_1 + N_2, \end{aligned}$$

where

3 $$\begin{aligned}  &   N_1 = S_A+E_A+I_A+S_r+E_r+I_r, \ \end{aligned}$$

4 $$\begin{aligned}  &   N_2= S_B+E_B+I_B+S_R+E_R+I_R. \end{aligned}$$

**Fig. 1:**
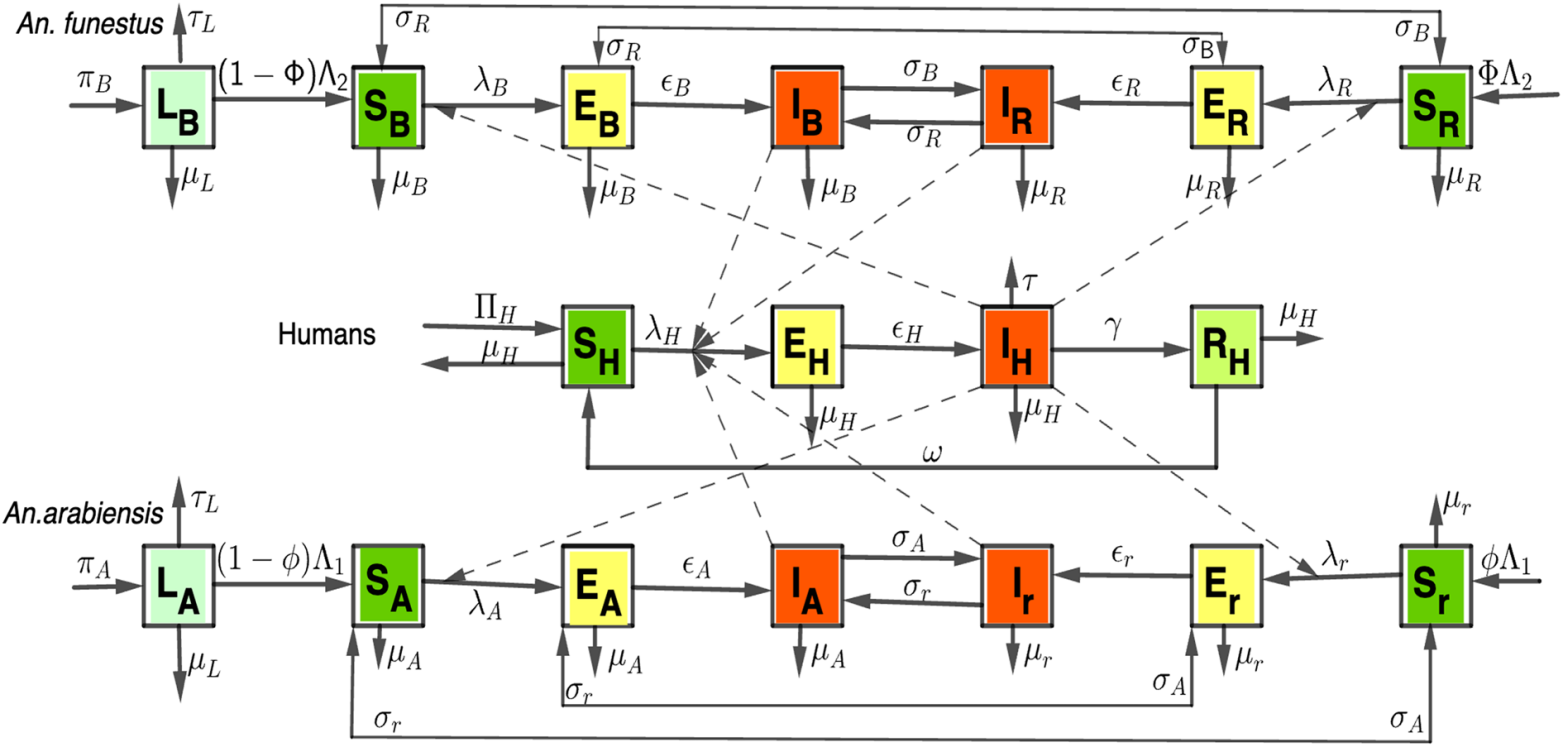
The flowchart depicting human-mosquito interactions: Infectious humans transmit malaria to susceptible mosquitoes, and infectious mosquitoes infect susceptible humans

Based on the flow diagram (Figure 1), the model is derived as a deterministic, non-autonomous system of nonlinear ordinary differential equations (9) (see Appendix 1).

In this model, the forces of infection $(\lambda _H, \lambda _A, \lambda _B, \lambda _R, \lambda _r)$ defined as gauge of the likelihood of malaria transmission from infectious human to susceptible mosquito and from infectious mosquito to susceptible human are defined using the concepts by Ngonghala et al. [32], and Ngwa et al. [34] by equations (5) and (6),

5 $$\begin{aligned}  &   \lambda _H = \dfrac{P_0(\beta _A I_A+\beta _B I_B+\beta _R I_R+\beta _r I_r)}{N_H}, \ \ \ \lambda _A = \dfrac{P_1 \beta _A I_H}{N_H}, \end{aligned}$$

6 $$\begin{aligned}  &   \lambda _B = \dfrac{P_1 \beta _B I_H}{N_H}, \ \ \ \lambda _r = \dfrac{P_1 \beta _r I_H}{N_H}, \ \ \ \lambda _R= \dfrac{P_1 \beta _R I_H}{N_H}. \end{aligned}$$

LLINs are generally highly effective when first obtained and after being impregnated with insecticides, but their effectiveness wanes over time. However, even in the absence of insecticide, nets serve as physical barriers that keep mosquitoes away from people, so the degree of protection is not entirely depleted [46].

**Fig. 2:**
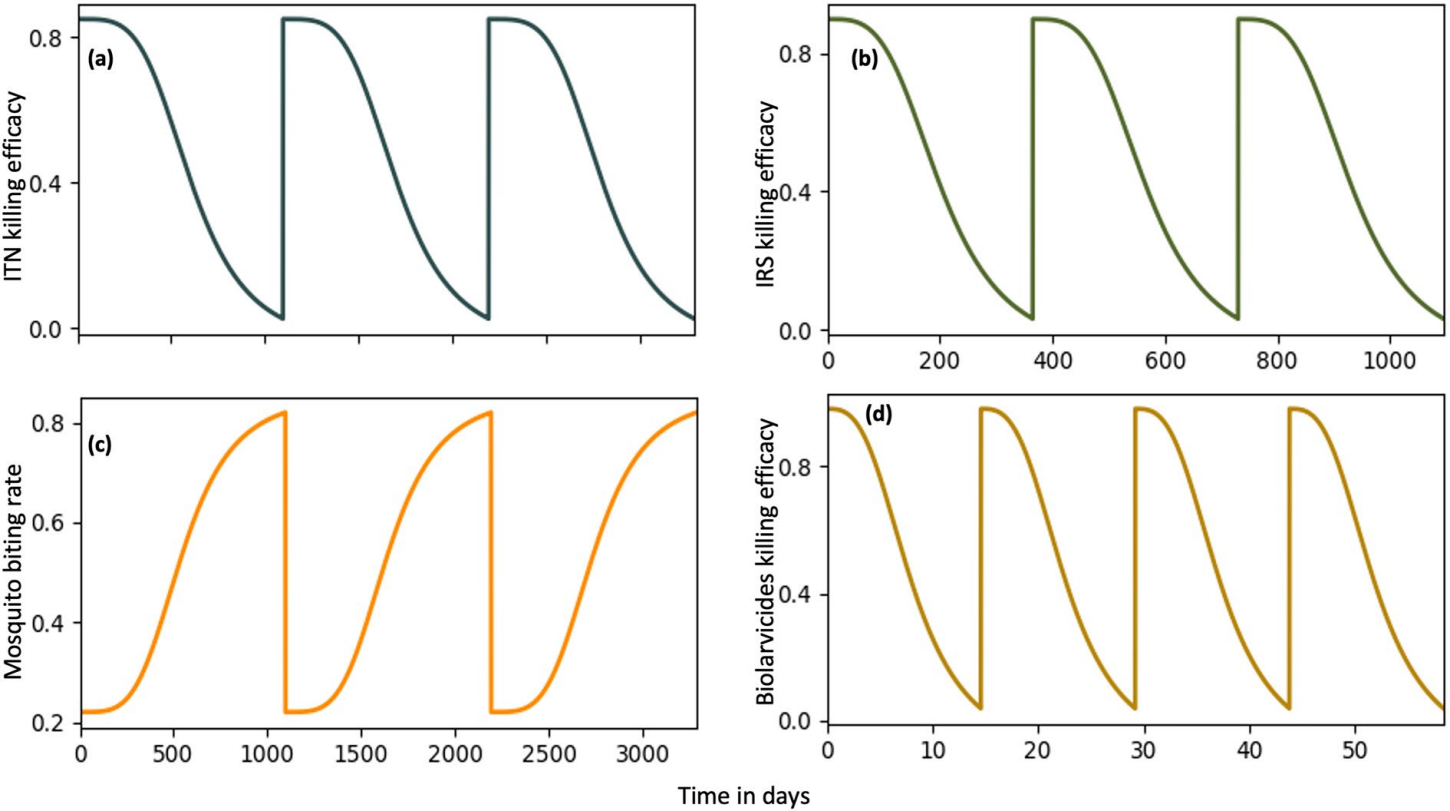
Graphical representation of the efficacy dynamics for: **a** ITNs, **b** IRS, and **d** biolarvicides. Initially, each vector control intervention demonstrates high efficacy, which wanes over time until the intervention is either retreated (particularly for ITNs), or replaced. **c** the mosquito biting rate, which is initially low following the implementation of ITNs, but increases as the insecticide efficacy wanes. **a** and **c** assume a usable life of ITNs of 3 years [12], while **b** and **d** assume durations of 1 year [49] for IRS and 14 days [50] for biolarvicides, respectively

The overall mortality and biting rates are modelled following Ngonghala et al. [32] and Ng’habi et al. [47], accounting for the time-dependent decline in the effectiveness of LLINs, IRS, and biolarvicides. It was assumed that the ITN-induced mortality does not always reach zero by the third year [48], retaining approximately 5% of their initial killing efficacy (Refer to Figure 2). Thus, the mortality and bite rates are respectively defined through the functions *f*(*t*) and $\beta (t)$ in equations (7) and (8),

7 $$\begin{aligned}&f_j(t) = \mu _{j0} + \Big (1 + \frac{1}{2^p}\Big ) \displaystyle \sum _{n=1}^{3} b_n \xi _{jn}\nonumber \\&\quad \left( \dfrac{1}{1 + \Big (\dfrac{1.8(t \bmod T_{n})}{T_{n}}\Big )^p} - \dfrac{1}{2^p + 1} \right) \end{aligned}$$

where the actual mortality $\mu _j$ is given by $\mu _j$ = $\dfrac{\exp f_j(t) }{ \exp (1+f_j(t) )}$,

8 $$\begin{aligned} \beta _{j} (t)= \beta _{j1} + \dfrac{( \beta _{j0} -\beta _{j1}) b_1 \xi _{j0}}{1+ \left( \dfrac{t \bmod T_1}{T_1} \right) ^p} \ \ \ \text {where} \ j\in (A,r,B, R). \end{aligned}$$

The parameters $b_1, b_2 \ \text {and} \ b_3$ represent the LLINs, IRS and biolarvicides coverages, while $\xi _{j1}$, $\xi _{j2}$ and $\mu _L$ denote their respective initial killing efficacies. Additionally, $\mu _{j0}$ stands for the natural death rates of insecticide susceptible and resistant mosquitoes. Moreover, the parameters $\beta _{j1}$ and $\beta _{j0}$ are correspondingly representing the maximum and minimum mosquito biting rates; these values indicate the highest and lowest feeding frequencies of mosquitoes under ideal conditions, leading to the biting rate $\beta (t)$ defined as the average number of mosquito bites a human experience daily, and is influenced by some factors such as ITN coverage and efficacy. In fact, the biting rate is crucial for evaluating disease transmission rates and the effectiveness of vector control tools. The parameter $p>1$ is the dimensionless shape constant, $T_{n}>0$ is the useful life or duration of LLINs, IRS, and biolarvicides and $\xi _{j0}$ is the initial efficacy of LLINs as personal protection against mosquitoes (capacity of LLINs to protect people beneath them from mosquito bites) and *n* is the integer representing the impact of each intervention on mosquito mortality. It is assumed that biolarvicide coverage is effective against both *An. funestus* and *An. arabiensis* populations. Although the deployment start times for ITN, IRS, and biolarvicides interventions are considered to differ, the model assumes the simultaneous distribution or replacement of all ITNs across households, alongside the concurrent deployment of IRS and biolarvicides, without incorporating seasonal variation. The temporal variations in killing efficacy of each vector control tool and mosquito biting rate are depicted by Figure 2.

### Intensity of resistance to pyrethroids

The WHO recognizes bioassays as a standard method for assessing phenotypic resistance in mosquitoes [7, 45]. These assays measure mosquito mortality after exposure to insecticides at concentrations equivalent to $1\times$, $5\times ,$ or $10\times$ the discriminating dose, with mortality rates corresponding to varying levels of resistance intensity: low, moderate, and high, respectively [21]. Pyrethroid resistance intensity in this study is quantified on a scale from 0 to 1, representing low (0.25), moderate (0.5), high (0.75), and highest (1) resistance intensities. Low resistance is defined as $\ge 98\%$ mortality at $5\times$ the discriminating dose, moderate as $<98\%$ mortality at $5\times$ but $\ge 98\%$ at $10\times$, high as $<98\%$ mortality at 10, and highest resistance (assumed) as $<98\%$ mortality at $15\times$ the discriminating dose. A resistance intensity of 0 indicates complete susceptibility of mosquitoes to the insecticide, thus, as resistance intensity increases, mosquito susceptibility to pyrethroids diminishes. It is important to note that resistance in this study refers specifically to pyrethroid resistance.

### Sensitivity analysis

**Fig. 3:**
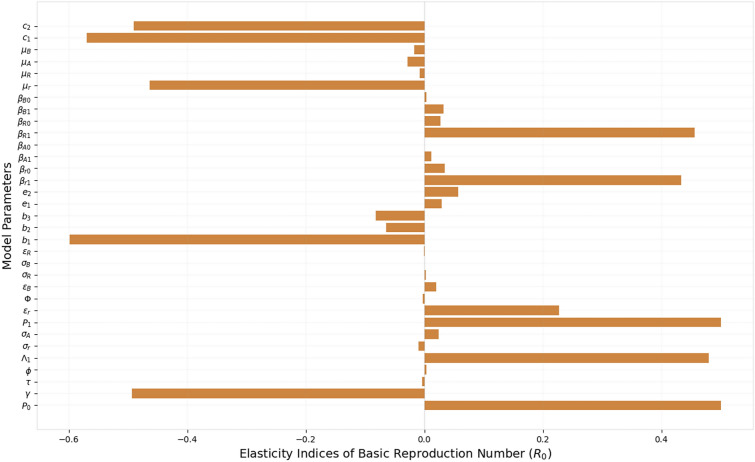
Sensitivity analysis of the important model parameters on reproduction number $(R_0)$. Parameters with larger elasticity indices have a greater effect on $R_0$; positive values increase it, while negative values decrease it

The sensitivity of the basic reproduction number ($R_0$) (see $R_0$ in Appendix 1) was evaluated using elasticity indices to identify the most influential parameters [51]. Positive indices indicate a direct relationship with $R_0$, while negative indices imply an inverse relationship. Key parameters influencing $R_0$ include ITN coverage ($b_1$), biting rates of resistant *An. funestus* ($\beta _{R1}$) and *An. arabiensis* ($\beta _{r1}$), immunization ($c_1$) and treatment coverage ($c_2$), human recovery rate ($\gamma$), transmission probabilities ($P_0$, $P_1$), mosquito recruitment ($\Lambda _1$), and mortality rates, all critically shaping malaria transmission dynamics (Figure 3). For instance, a 10% increase in $\beta _{R1}$ and $\beta _{r1}$ raises $R_0$ by 4.6% and 4.3%, respectively, while a 10% increase in $P_0$ or $P_1$ raises $R_0$ by 5%. Conversely, a 10% increase in $b_1$ and $c_1$ reduces $R_0$ by 6% and 5.7%, respectively. These sensitivities highlight the critical need to manage malaria by reducing mosquito biting and transmission rates while enhancing ITN, immunization, and treatment coverage, and emphasize the substantial role of insecticide resistance in sustaining transmission.

### Model analysis, interventions and simulated scenarios

The stability analysis of the equilibrium point for model (9) is presented in Appendix 1. The model was simulated using Python to investigate how mosquito biting rates, reproduction numbers, and mortality respond to varying levels of ITN, IRS, and biolarvicide coverage under different resistance intensities. Additionally, the impact of treatment and vaccination on malaria reduction in Tanzania was evaluated. The simulations focused on the roles of pyrethroid-PBO ITNs, organophosphate-based IRS, and biolarvicides as key tools of current strategy to combat insecticide resistance in Tanzania [24], as well as artemisinin-based combination therapy (ACT) as primary antimalarial drugs (assumed to have a constant efficacy of $98.5\%$) [52], and the WHO-approved malaria vaccines, RTS,S/AS01 and R21/Matrix-M (all assumed to reduce malaria symptomatic cases in children by 75%) [53, 54]. In this study, vaccination is assumed to target children under five. To reflect this age group, which constitutes 15.4% of the total population in Tanzania [55], effective vaccination coverage is defined as 0.154 times the total coverage level. Furthermore, given the critical role of infected mosquitoes and humans in malaria transmission, our analysis only focused on how the infected groups (Figure 1) respond to interventions.

**Table 1:** The description of state variables for model system (9)

Variable	Description
$S_H$	Susceptible human population
$E_H$	Exposed human population
$I_H$	Infectious human population
$R_H$	Recovered human population
$L_A$	Immature *An. arabiensis* mosquitoes
$S_A$	Non-resistant *An. arabiensis* mosquitoes (susceptible to malaria parasites)
$E_A$	Non-resistant *An. arabiensis* mosquitoes (exposed to malaria parasites)
$I_A$	Non-resistant *An. arabiensis* mosquitoes (infectious)
$S_r$	Resistant *An. arabiensis* mosquitoes (susceptible to malaria parasites)
$E_r$	Resistant *An. arabiensis* mosquitoes (exposed to malaria parasites)
$I_r$	Resistant *An. arabiensis* mosquitoes (infectious)
$L_B$	Immature *An. funestus* mosquitoes
$S_B$	Non-resistant *An. funestus* mosquitoes (susceptible to malaria parasites)
$E_B$	Non-resistant *An. funestus* mosquitoes (exposed to malaria parasites)
$I_B$	Non-resistant *An. funestus* mosquitoes (infectious)
$S_R$	Resistant *An. funestus* mosquitoes (susceptible to malaria parasites)
$E_R$	Resistant *An. funestus* mosquitoes (exposed to malaria parasites)
$I_R$	Resistant *An. funestus* mosquitoes (infectious)

**Table 2:** Model parameters for model system (9) with their baseline values and ranges, Est.=Estimated

Parameter	Description	Baseline Value [Range]	Source(s)
$\Pi _H$	Human recruitment rate (day$^{-1}$)	0.046 [3.65, 9.13]$\times 10^{-2}$	Est. from [56]
$\Lambda _1$	*An. arabiensis* recruitment rate (day$^{-1}$)	0.79 [0.77, 0.87]	[57]
$\Lambda _2$	*An. funestus* recruitment rate (day$^{-1}$)	0.85 [0.73, 0.96]	Est. from [58]
$\sigma _A$	Mutation rate of non-resistant *An. arabiensis* (day$^{-1}$)	0.02 [0,1]	[59]
$\sigma _r$	Mutation rate of resistant *An. arabiensis* (day$^{-1}$)	0.04 [0,1]	[60, 61]
$\sigma _B$	Mutation rate of non-resistant *An. funestus* (day$^{-1}$)	0.01 [0,1]	Varies
$\sigma _R$	Mutation rate of resistant *An. funestus* (day$^{-1}$)	0.05 [0,1]	Varies
$\epsilon _H$	Rate at which exposed humans become infectious (day$^{-1}$)	0.071 [6.7, 20] $\times 10^{-2}$	[62, 63]
$\epsilon _A, \epsilon _B$	Infectiousness rate of non-resistant mosquitoes (day$^{-1}$)	0.1 [0.029, 0.33]	[63, 64]
$\epsilon _r, \epsilon _R$	Infectiousness rate of resistant mosquitoes (day$^{-1}$)	0.1 [0.029, 0.33]	[63, 64]
$\beta _{A1}$	Maximum biting rate of non-resistant *An. arabiensis* (day$^{-1}$))	0.5 [7.6, 75]$\times 10^{-2}$	[65, 66]
$\beta _{A0}$	Minimum biting rate of non-resistant *An. arabiensis* (day$^{-1}$)	0.07 [7.6, 75]$\times 10^{-2}$	[65, 66]
$\beta _{r1}$	Maximum biting rate of resistant *An. arabiensis* (day$^{-1}$)	0.6 [7.6, 75]$\times 10^{-2}$	[65, 66]
$\beta _{r0}$	Minimum biting rate of resistant *An. arabiensis* (day$^{-1}$)	0.075 [7.6, 75]$\times 10^{-2}$	[65, 66]
$\beta _{B1}$	Maximum biting rate of non-resistant *An. funestus* (day$^{-1}$)	0.5 [7.6, 75]$\times 10^{-2}$	[65, 66]
$\beta _{B0}$	Minimum biting rate of non-resistant *An. funestus* (day$^{-1}$)	0.07 [7.6, 75]$\times 10^{-2}$	[65, 66]
$\beta _{R1}$	Maximum biting rate of resistant *An. funestus* (day$^{-1}$)	0.7 [7.6, 75]$\times 10^{-2}$	[65, 66]
$\beta _{R0}$	Minimum biting rate of resistant *An. funestus* (day$^{-1}$)	0.075 [7.6, 75]$\times 10^{-2}$	[65, 66]
$\tau$	Human disease induced death rate (day$^{-1}$)	0.00009 [0, 4.1]$\times 10^{-4}$	[51]
$\omega$	The rate at which the recovered human lose immunity (day$^{-1}$)	0.00055 [55,110] $\times 10^{-4}$	[51]
$\gamma$	Humans recovery rate (day$^{-1}$)	0.0035 [1.4, 17]$\times 10^{-3}$	[62, 67]
$\mu _H$	Human natural death rate (day$^{-1}$)	0.000041 [3.3, 5.5] $\times 10^{-5}$	[68]
$\mu _{A0}$	Non-resistant *An. arabiensis’* natural death rate (day$^{-1}$)	0.24 [1.5, 34]$\times 10^{-1}$	[69]
$\mu _{r0}$	Resistant *An. arabiensis’* natural death rate (day$^{-1}$)	0.025 [1.2, 14]$\times 10^{-2}$	[40]
$\mu _{B0}$	Non-resistant *An. funestus’* natural death rate (day$^{-1}$)	0.14 [1.5, 34]$\times 10^{-1}$	[69]
$\mu _{R0}$	Resistant *An. funestus’* natural death rate (day$^{-1}$)	0.02 [1.2, 14]$\times 10^{-2}$	Assumed
$P_0$	Mosquito to human malaria transmission probability	0.018 [0, 1]	[70]
$P_1$	Human to mosquito malaria transmission probability	0.28 [0, 1]	[32]
$\phi$	Fraction of recruited *An. arabiensis* with resistance	0.02 [0,1]	Est. from [57, 71]
$\Phi$	Fraction of recruited *An. funestus* with resistance	0.2 [0,1]	Est. from [58, 71]
$b_1$, $b_2$, $b_3$	ITN, IRS and biolarvicides coverage respectively	0-1	Varies
$c_1$, $c_2$	Immunization and treatment coverage respectively	0-1	Varies
$\pi _A$	Immature *An. arabiensis* hatching rate (day$^{-1}$)	0.325 [0, 1]	[58]
$\pi _B$	Immature *An. funestus* hatching rate (day$^{-1}$)	0.325 [0, 1]	[58]
$\mu _L$	Immature mosquito natural death rate (day$^{-1}$)	0.2 [0. 1]	Varies
$\tau _L$	Biolarvicides initial killing efficacy	0.98	[50]
$\xi _{A0}$	ITNs initial killing efficacy against susceptible *An. arabiensis*	1	[72]
$\xi _{r0}$	ITNs initial killing efficacy against resistant *An. arabiensis*	0.753	[71]
$\xi _{B0}$	ITNs initial killing efficacy against susceptible *An. funestus*	1	[72]
$\xi _{R0}$	ITNs initial killing efficacy against resistant *An. funestus*	0.644	[73]
$\xi _{A1}$	IRS initial killing efficacy against susceptible *An. arabiensis*	1	[72]
$\xi _{r1}$	IRS initial killing efficacy against resistant *An. arabiensis*	0.976	[74]
$\xi _{B1}$	IRS initial killing efficacy against susceptible *An. funestus*	1	[66]
$\xi _{R1}$	IIRS initial killing efficacy against resistant *An. funestus*	1	[66]
$T_1, T_2, T_3$	Useful lifetime of ITN, IRS and Biolarvicides (days)	3 $\times$ 365, 365, 14	[12, 49, 50]
$e_1$, $e_2$	*An. arabiensis* and *An. funestus* resistance intensities	[0,1]	Varies

## Results

**Fig. 4:**
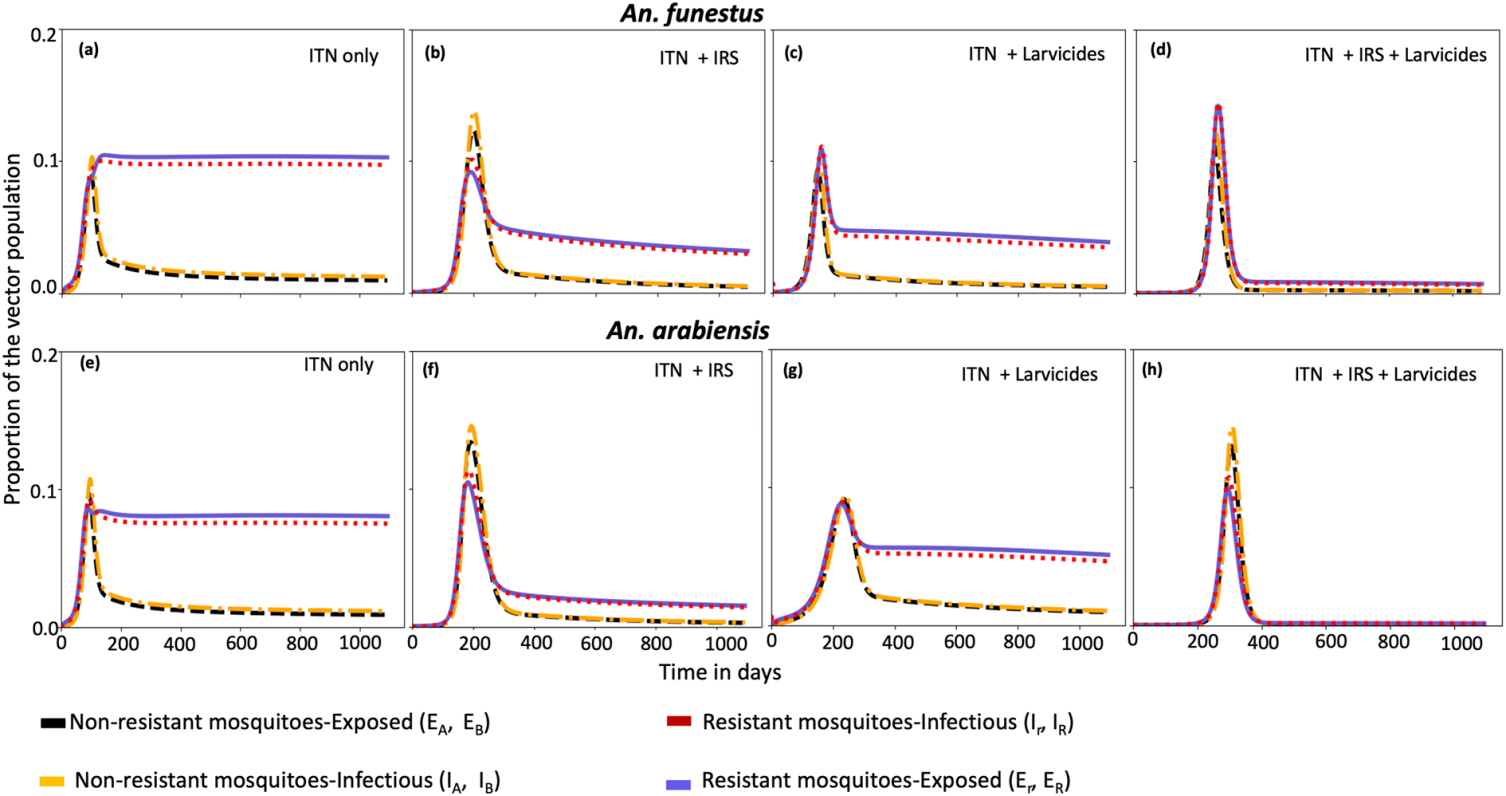
The impact of pyrethroid-PBO ITNs, organophosphate-based IRS, and biolarvicides on the densities of susceptible and resistant *An. funestus* and *An. arabiensis* at moderate resistance intensity (50%), with each intervention at 80% coverage

Figure 4 illustrates the impact of combining vector control tools on resistant and susceptible mosquito populations that are moderately resistant (50% resistance intensity). At 80% coverage, pyrethroid-PBO ITNs slightly increase mortality of resistant *An. arabiensis* compared to resistant *An. funestus* (Figure 4(a) and (e)). Adding IRS at 80% coverage to ITNs reduced the proportion of infected resistant *An. funestus* and *An. arabiensis* by 57.68% and 68.12%, respectively (Figure 4(b) and (f)). Meanwhile, combining biolarvicides with ITNs at the same coverage led to a 43.86% and 34.87% reduction in infected resistant *An. funestus* and *An. arabiensis*, respectively (Figure 4(c) and (g)). These results, however, are based on the parameter values in Table 2 and the assumptions of the proposed model and should not be broadly generalized. They should also be interpreted in terms of the proportion of mosquitoes killed rather than absolute numbers, due to species-specific feeding behaviours.

**Fig. 5:**
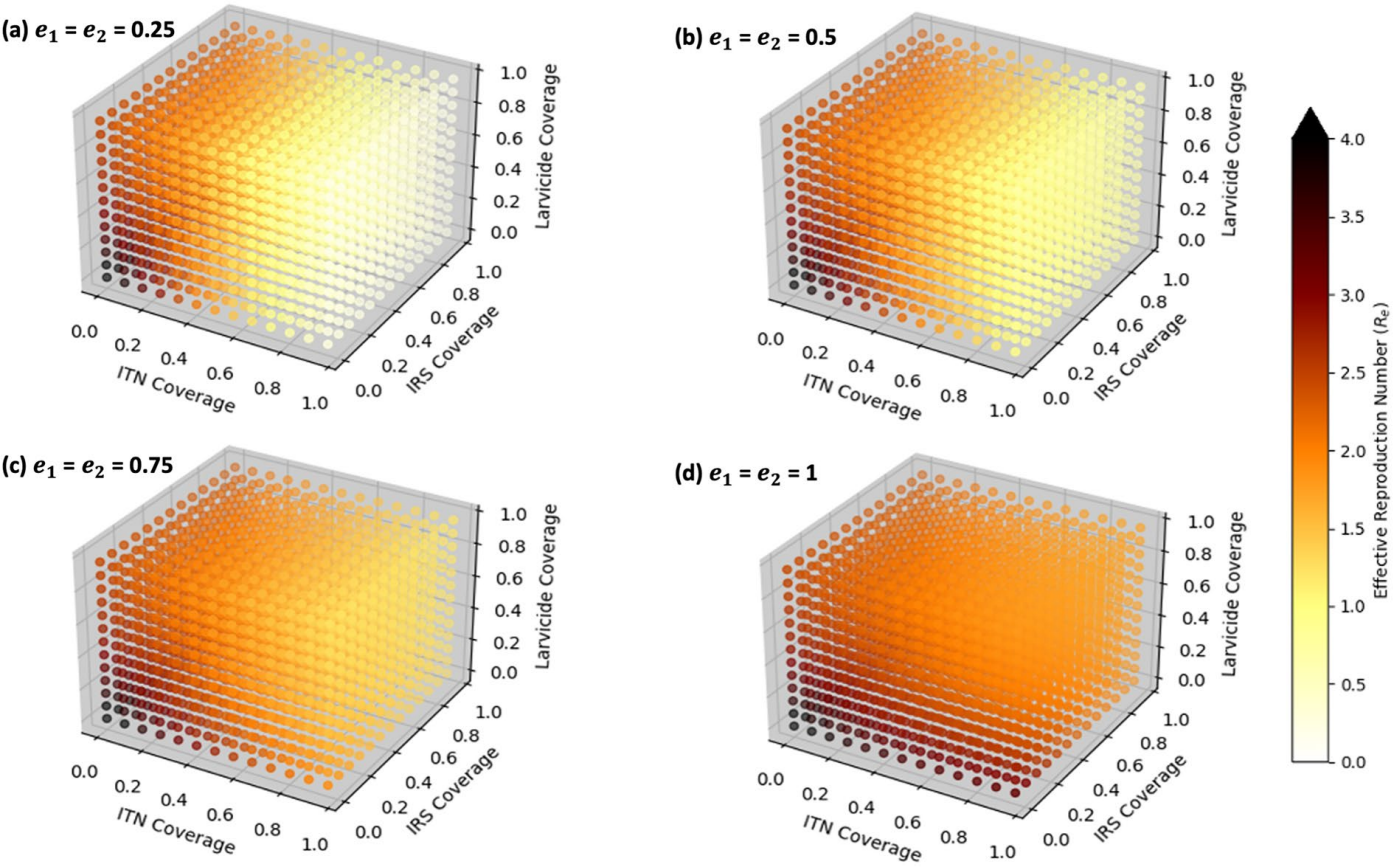
Impact of combination of pyrethroid-PBO ITNs, organophosphate-based IRS and biolarvicides on effective reproduction number under different resistance intensities ($e_1$, $e_2$ are *An. arabiensis* and *An. funestus* resistance intensities, respectively.)

Figure 5 shows the correlation between resistance intensity and the effective reproduction number ($R_e$). At low intensities of resistance (25%), a combination of at least 60% ITN and IRS coverage, along with 30% biolarvicide coverage, reduced $R_e$ to below 0.5 (Figure 5 (a)). At 50% resistance, the same strategy lowers $R_e$ to between 0.5 and 1 (Figure 5 (b)), but when resistance reaches 75% or higher, even this combination of three methods fails to reduce $R_e$ below 1 (Figure 5 (c)). At maximum-intensities of pyrethroid resistance (100%), while the ITNs can still provide some community protection, $R_e$ ranges from 1.5 to 2.5 even with 100% coverage of the ITNs (Figure 5 (d)).

**Fig. 6:**
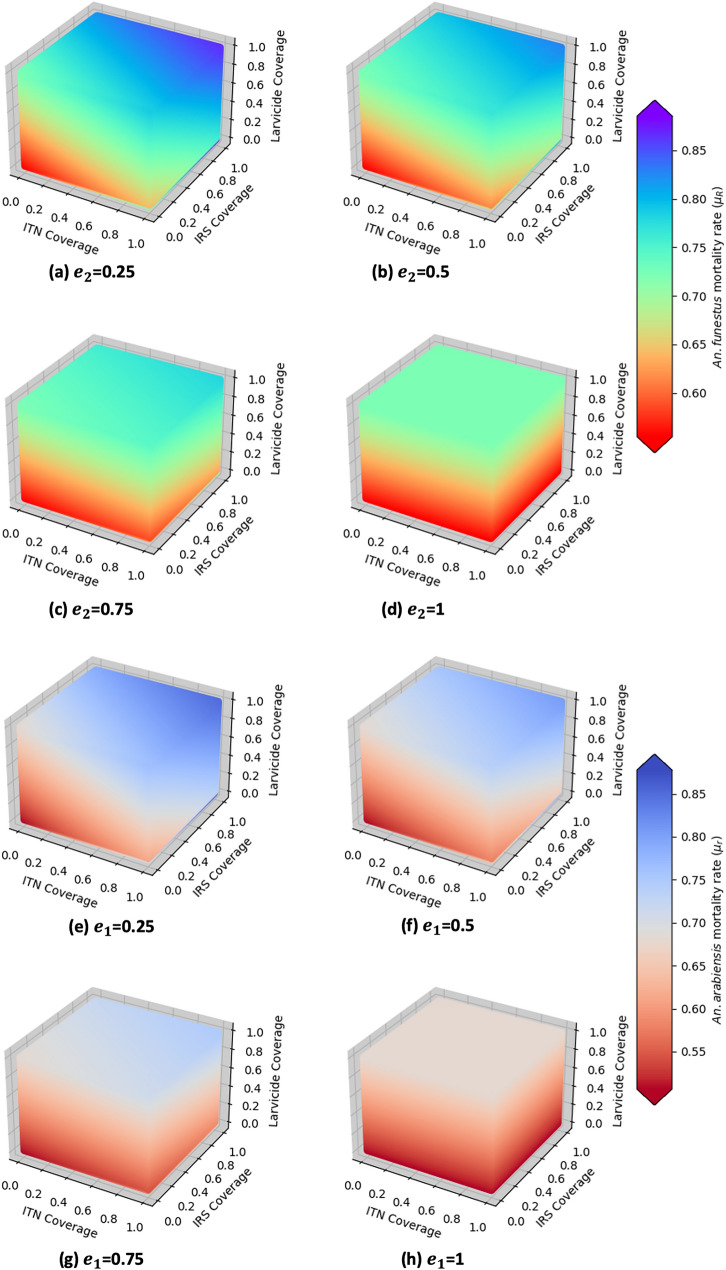
Impact of combination of pyrethroid-PBO ITNs, organophosphate-based IRS and biolarvicides on mortality of malaria vectors under different resistance intensities ($e_1$, $e_2$ are *An. arabiensis* and *An. funestus* resistance intensities respectively.)

Figure 6 illustrates the inverse relationship between mosquito mortality rates and resistance intensities for *An. funestus *and *An. arabiensis*. At low resistance intensities of 25%, a combination of at least 60% ITN and IRS coverage, along with 30% biolarvicide coverage, can achieve over 70% mosquito mortality 6(a) and (e)). However, when the resistance intensity exceeds 75%, the mortality drops below 60%. Since biolarvicides target immature mosquito stages, they remain effective even at 100% resistance, with at least 60% biolarvicide coverage still greatly reducing mosquito density 6(c),(d),(g) and (h)).

**Fig. 7:**
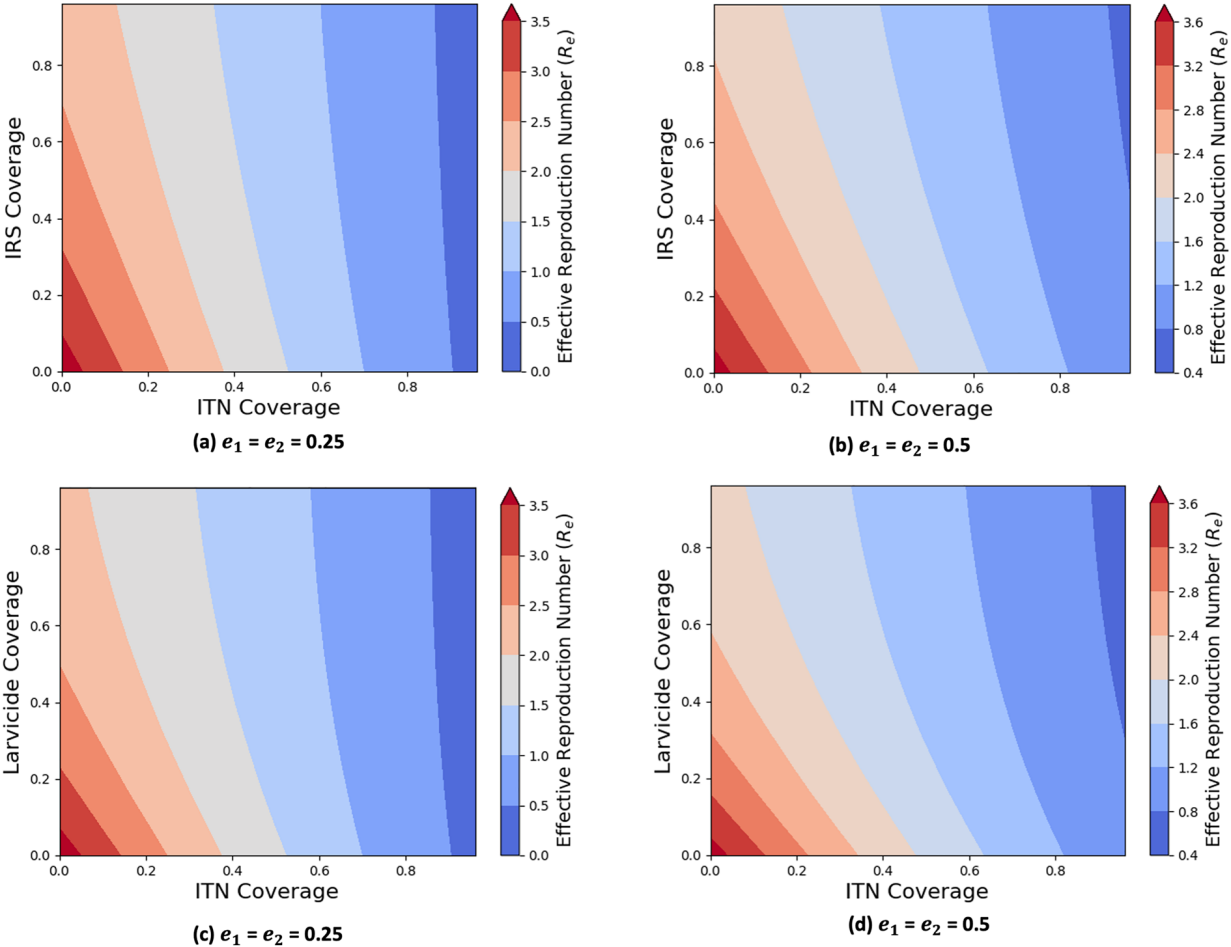
Impact of combining pyrethroid-PBO ITNs with organophosphate-based IRS or biolarvicides on effective reproduction number ($e_1$, $e_2$ denote *An. arabiensis* and *An. funestus* resistance intensities, respectively.)

**Fig. 8:**
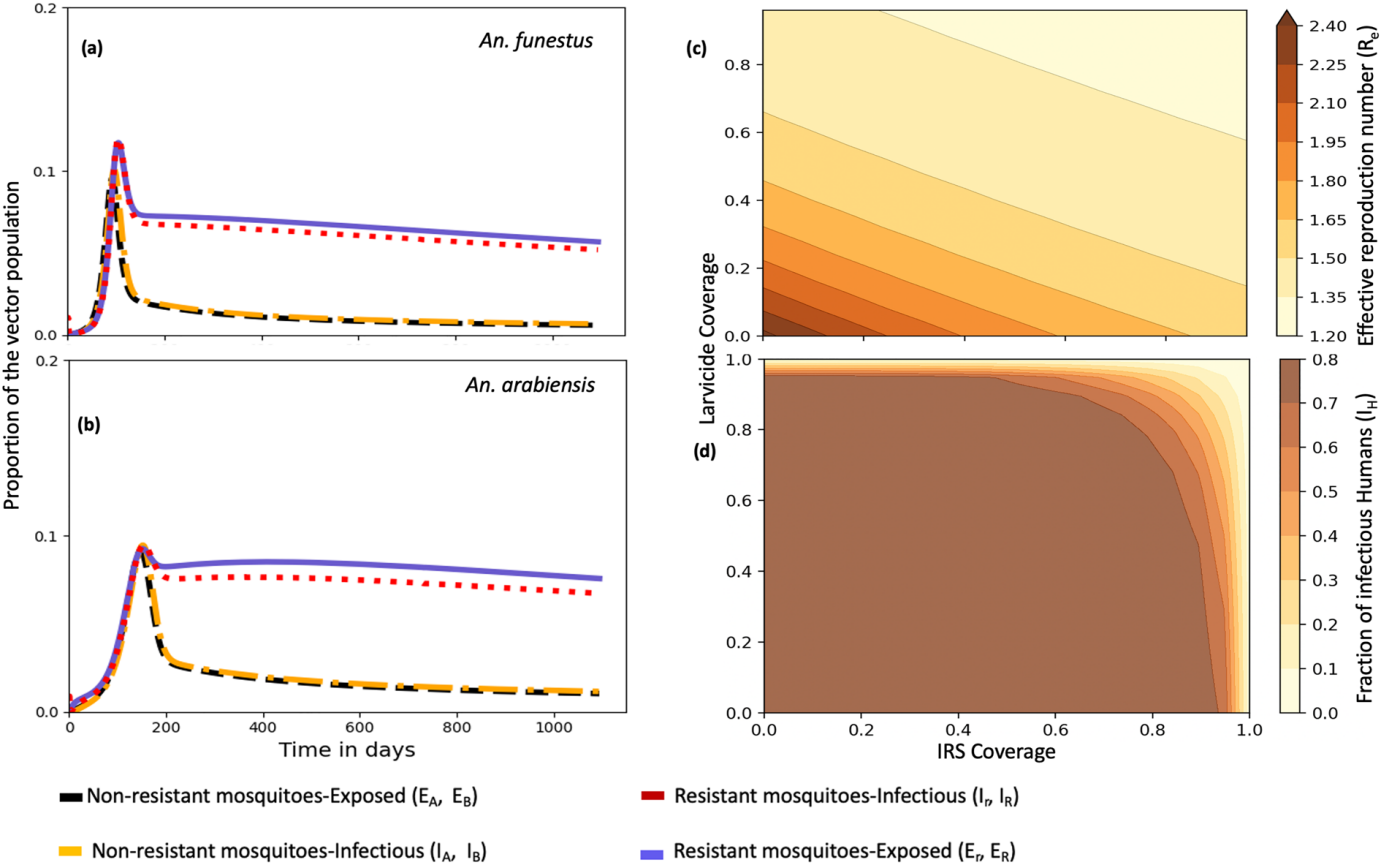
Impact of combining IRS with biolarvicides at zero ITN coverage: **a, b** resistant and non-resistant infected *An. funestus* and *An. arabiensis* (each intervention at 80% coverage); **c** effective reproduction number ($R_e$); **d** fraction of infectious humans

Figure 7 illustrates the impact of combining pyrethroid-PBO ITNs with IRS or biolarvicides on the effective reproduction number $R_e$ at low (25%) and moderate (50%) intensities of resistance. Under low intensities of resistance, ITN coverage of at least 70% can bring $R_e$ below 1. However, reducing $R_e$ to below 0.8 requires either 61% ITN coverage with 81% IRS coverage (Figure 7(a)) or 61% ITN coverage with 56% biolarvicide coverage (Figure 7(c)). At moderate resistance level, achieving a reduction in $R_e$ to below 1 requires either at least 71% ITN coverage and 50% IRS coverage (Figure 7(b)) or at least 71% ITN coverage and 32% biolarvicide coverage (Figure 7(d)). In contrast, Figure 8 presents the impact of combining IRS and biolarvicides without ITNs. The 90% coverage with both interventions fails to reduce $R_e$ below 1, as ITNs provide direct personal protection and reduce human-vector contact (Figure 8(c)). Moreover, 80% coverage with IRS and/or biolarvicides alone is insufficient to lower the proportion of infected individuals below 50% (Figure 8(d)), highlighting the crucial role of ITNs in integrated malaria control.

**Fig. 9:**
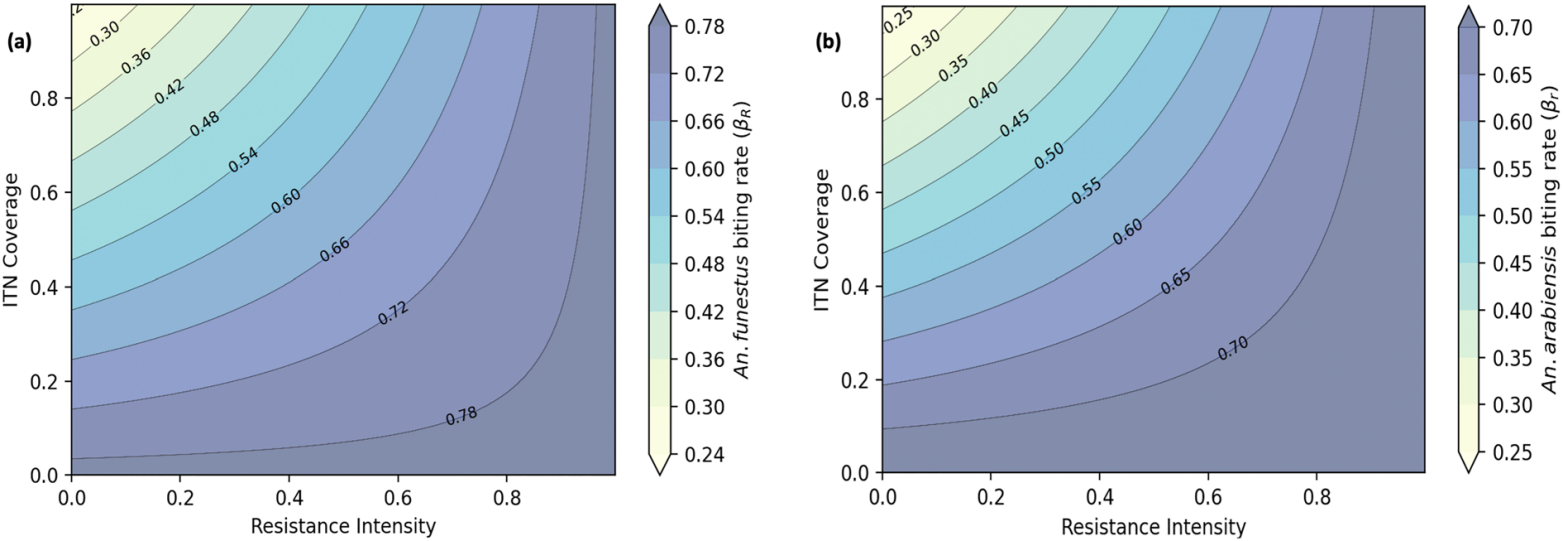
Impact of pyrethroid-PBO ITN coverage and resistance intensities on biting rates: **a** *An. funestus*, **(b)** *An. arabiensis*

Figure 9 illustrates how varying ITN coverage levels affect mosquito biting rates under different resistance intensities. With at least 80% ITN coverage, the biting rates of *An. funestus * and *An. arabiensis* can be reduced to below 0.3 and 0.2 bites/mosquito/day, respectively, as long as resistance intensity remains below 50% (Figure 9 (a) and (b)). However, when resistance intensity exceeds 50%, ITN effectiveness declines greatly, leading to biting rates above 50%, with resistant *An. funestus* exhibiting higher biting rates than resistant *An. arabiensis*.

**Fig. 10:**
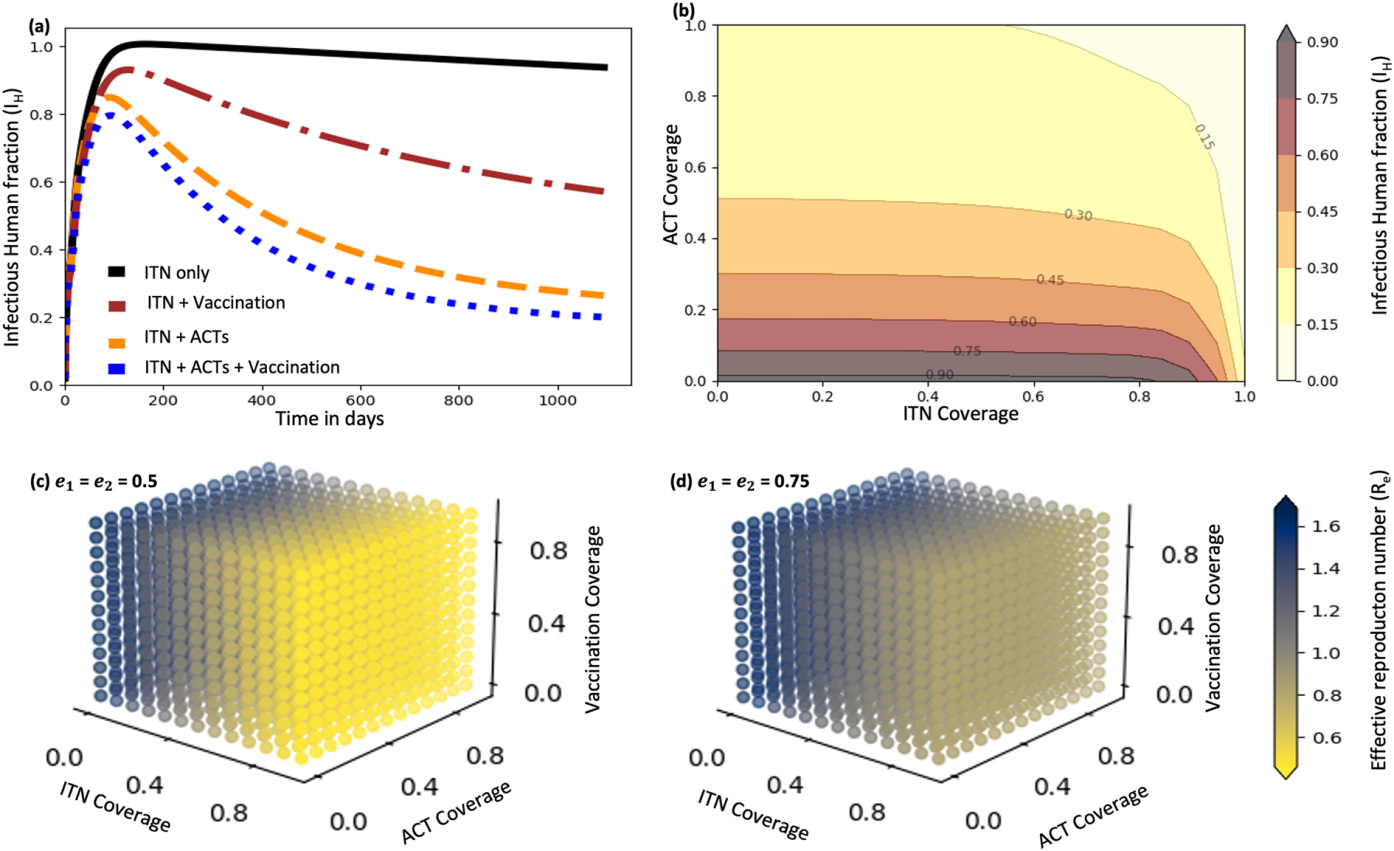
Impact of combining ITNs with ACT and/or vaccination on infectious humans ($I_H$) and effective reproduction number ($R_e$): **a** all interventions at 80% coverage at moderate resistance (0.5); **b** varying ITN and ACT coverage with vaccination fixed at 80% at moderate resistance (0.5); **c, d** varying coverages at moderate (0.5) and high (0.75) resistance levels ($e_1$, $e_2$ are *An. arabiensis* and *An. funestus* resistance intensities, respectively)

Lastly, Figure 10 underlines the necessity of an integrated malaria control approach combining ITNs, effective treatment, and immunization to improve public health outcomes. The model indicates that ITNs alone slightly reduce the proportion of infectious individuals. However, adding vaccination at 80% coverage (estimated to target children under five, who represent 15.4% of entire population [55]), reduces the infectious proportion to below 60% at the end of third year. Adding ACT to ITN (each at 80% coverage) reduces this proportion to below 30% at moderate pyrethroid resistance (Figure 10(a)). Similarly, a combined strategy of at least 80% coverage for ITNs, treatment, and immunization achieves a reduction of infectious proportion to below 20% (Figure 10(a) and (b)). Furthermore, at moderate pyrethroid resistance (50%), at least 40% ITN coverage combined with 80% ACT and 40% vaccination coverage reduces $R_e$ below 1. At high resistance level (75%), increasing ITN coverage to 80% while maintaining the same ACT and vaccination levels also reduces $R_e$ below 1 (Figure 10(c) and (d)).

## Discussion

In Tanzania, *An. funestus* and *An. arabiensis* are the predominant malaria vectors, and both have widespread resistance to pyrethroids [75, 76]. Although *An. arabiensis* typically surpasses *An. funestus* in prevalence across many regions [75, 76], *An. funestus* has consistently shown greater vectorial efficiency, with higher contributions to entomological inoculation rate (EIR), even in areas where *An. arabiensis* is more abundant [66, 71, 75, 77]. Moreover, *An. funestus* exhibits greatly higher insecticide resistance at equivalent exposure levels [23, 78].

This study utilized an innovative mathematical model to examine the relationship between insecticide resistance intensities and the performance of the main vector control tools, when used alone or in combination, under varying resistance intensities.

Although pyrethroid-PBO ITNs alone confer some community protection, their efficacy diminishes over time and with increasing insecticide resistance intensities [79]. The marked restoration of susceptibility in pyrethroid-resistant *An. arabiensis* compared to *An. funestus* following pre-exposure to the synergist PBO [78] highlights the reduced effectiveness of PBO bednets in regions dominated by *An. funestus*. Consistent with findings on the constrained efficacy of pyrethroid-PBO ITNs against both *An. funestus* and *An. arabiensis*, the results further emphasize the added public health value of combining ITNs with IRS and/or biolarvicides to achieve more substantial reductions in mosquito densities and malaria transmission. The important observation from this study demonstrates the effectiveness of biolarvicides in settings with substantial insecticide resistance.

While combining interventions such as ITNs and IRS are emphasized in high-transmission and resistance settings [12, 80–82], locally tailored integrated vector control strategies remain critical to maximize impact [83]. Our study demonstrates that, the combination of vector control interventions with different mode of actions is essential in significant reducing the transmission in situations with low to moderate resistance levels. Unfortunately, at high resistance levels, transmission reduction remains limited. The study further highlights that combining vector control with other interventions such as immunization can significantly reduce transmission even in areas with high resistance levels. These findings underscore the need for adaptive, locally informed intervention strategies that account for both epidemiological context and resistance intensity.

Following the recent discontinuation of IRS in Tanzania [25], and the logistical and financial challenges of deploying both IRS and ITNs [12], this study provides valuable insights for the NMCP to consider integrating biolarvicides with ITNs as a viable alternative. Larval source management (LSM), particularly through biolarvicides, has also demonstrated effectiveness across diverse settings and is increasingly supported by communities [84], making it a promising complementary strategy. The growing recommendation to adopt LSM as a core vector control intervention reflects the urgent need to revitalize efforts amid stalled progress against malaria in Africa [85]. Since biolarvicides target immature mosquito stages and remain effective regardless of species, behavioral differences, or insecticide resistance levels, our study demonstrates their consistent efficacy. However, their effectiveness relies on enhancing community engagement and using local labor to support efficient and sustainable implementation [84, 85]. Further research is needed to assess their long-term impact, short residual activity, and large scale deployment, particularly given their limited use in urban and arid regions where habitats are few, fixed, and findable [12].

Unfortunately, the study also demonstrates that, despite the efficacies of IRS and biolarvicides, malaria transmission cannot be effectively reduced without the use of ITNs. Specifically, the findings demonstrate that with zero ITN coverage, the combined use of IRS and biolarvicides is insufficient to reduce the reproduction number below 1 even at low mosquito resistance intensity. Hence, integrating ITNs with an additional vector control tool that uses insecticides with different modes of action can enhance the overall impact against malaria transmission and lower the resistance selection pressure [80, 86]. Consequently, the simulated findings additionally demonstrate that the combination of ITNs, IRS and biolarvicdes yields the most effective outcomes in diminishing the densities of resistant mosquitoes and their reproduction numbers, even when mosquito resistance to ITNs intensifies.

This study also highlights the importance of effective treatment and immunization alongside vector control measures, advocating for comprehensive, integrated approaches to achieve malaria elimination goals. In reality, no single magic bullet is sufficient to eliminate malaria burden in any endemic country [87]. However, putting into practice a complete package of locally appropriate interventions with adequate coverage may help to lower the malaria burden [88], hence helping to achieve the GTS goal of a 90% reduction in malaria incidence and mortality by 2030 [11, 89]. In light of the recent approval and growing adoption of the RTS,S/AS01 and R21/Matrix-M malaria vaccines [54, 90], and WHO recommendations supporting their integration with interventions such as ITNs [53], this study emphasizes the critical need for greater investment in multiple intervention groups to effectively mitigate the malaria burden.

The current findings support the hypothesis that insecticide resistance can substantially impede global malaria control and elimination efforts, aligning with prior research and WHO reports highlighting this growing threat [12, 23, 36, 37, 39, 78, 80, 91–93]. The model presented in this study can be modified and utilized by others to investigate the impact of combining interventions under different local conditions, provided that the underlying assumptions are considered and the parameters are adjusted to reflect the specific setting and dominant vector species. Therefore, generalizing these findings without careful evaluation may lead to inappropriate conclusions or policy recommendations. Rather than serving as universal guidance, the study spotlights the necessity for supplementary intervention tools such as biolarvicides, particularly in regions where malaria transmission is predominantly driven by both *An. funestus *and *An. arabiensis*, as well as the need for malaria case management initiatives. Despite its novelty, the study addresses only phenotypic resistance, excluding genotypic mechanisms, antimalarial drug resistance, variations in the timing and distribution of interventions and the time-dependent decline in the effectiveness of ACT and vaccines. It also models only *An. funestus* and *An. arabiensis*, ignoring *An. gambiae s.s.*, which contributes less than 25% to the average annual EIR [75]. Including all three primary vectors would improve model accuracy, particularly in areas where *An. gambiae s.s.* predominates. Future work should address these gaps and incorporate mosquito allele frequencies to better reflect resistance dynamics.

## Conclusions

This modelling study demonstrates that insecticide resistance greatly diminishes the effectiveness of vector control tools. However, combining ITNs with IRS and/or biolarvicides improves transmission reduction at moderate resistance levels, but remains inadequate under high resistance intensities. While biolarvicides play a crucial role by targeting immature mosquitoes and remaining effective even under high resistance, they cannot substantially reduce malaria transmission without ITNs, underscoring the need for their integration with other vector control tools. Integrating immunization and effective case management with ACT further strengthens control efforts, reducing transmission and infectious individuals. Effective malaria control in high-resistance settings therefore requires a tailored approach that combines vector control strategies with additional interventions, such as immunization and treatment, adapted to local resistance patterns and dominant vector behaviours.

## Data Availability

The data supporting this study are from previously reported studies as listed and cited in Table 2.

## Appendix 1

Model equations derived from schematic diagram 1:

9 $$\begin{aligned} {\left\{ \begin{array}{ll} \dot{S_H} &  = \Pi _H + \omega R_H -( \mu _H + P_0(\beta _A I_A+\beta _B I_B+ \beta _R I_R+ \beta _r I_r)) S_H \\ \\ \dot{E_H}& = P_0(\beta _A I_A+\beta _B I_B+ \beta _R I_R+ \beta _r I_r) S_H -(\epsilon _H +\mu _H) E_H\\ \\ \dot{I_H} & =\epsilon _H E_H -(\tau +\gamma + \mu _H) I_H \\ \\ \dot{R_H} &  = \gamma I_H-(\omega + \mu _H) R_H\\ \\ \dot{L_A}& = \pi _A-(\tau _L+\mu _L) L_A- (1-\phi ) \Lambda _1 L_A \\ \\ \dot{S_A}& = (1-\phi ) \Lambda _1+ \sigma _r S_r- (\mu _A+\sigma _A+ P_1 \beta _A I_H) S_A\\ \\ \dot{E_A}& = P_1 \beta _A I_H S_A+ \sigma _r E_r - (\epsilon _A +\mu _A+ \sigma _A) E_A\\ \\ \dot{I_A}& =\epsilon _A E_A + \sigma _r I_r - (\mu _A+\sigma _A) I_A\\ \\ \dot{S_r} & = \phi \Lambda _1 + \sigma _A S_A - (\mu _r + \sigma _r +P_1 \beta _r I_H) S_r \\ \\ \dot{E_r}& =P_1 \beta _r I_H S_r + \sigma _A E_A -(\epsilon _r +\mu _r+\sigma _r) E_r\\ \\ \dot{I_r}& = \epsilon _r E_r +\sigma _A I_A -(\mu _r + \sigma _r) I_r\\ \\ \dot{L_B}& = \pi _B-(\tau _L+\mu _L) L_B- (1-\Phi ) \Lambda _2 L_B \\ \\ \dot{S_B}& = (1-\Phi ) \Lambda _2 + \sigma _R S_R- (\mu _B + \sigma _B+P_1 \beta _B I_H) S_B\\ \\ \dot{E_B}& =P_1 \beta _B I_H S_B + \sigma _R E_R- (\mu _B +\sigma _B+ \epsilon _B ) E_B\\ \\ \dot{I_B}& =\epsilon _B E_B + \sigma _R I_R -(\mu _B+ \sigma _B) I_B\\ \\ \dot{S_R}& = \Phi \Lambda _2 + \sigma _B S_B- (\mu _R+\sigma _R + P_1 \beta _R I_H) S_R\\ \\ \dot{E_R}& =P_1 \beta _R I_H S_R +\sigma _B E_B- (\mu _R +\sigma _R+ \epsilon _R ) E_R\\ \\ \dot{I_R}& =\epsilon _R E_R + \sigma _B I_B - (\mu _R+\sigma _R) I_R \end{array}\right. } \end{aligned}$$

### Existence, positivity and boundedness of the solutions

All the state variables and parameters of the proposed model (9) are non-negative because they represent human and mosquito populations. We show that our model is epidemiologically and mathematically well-defined in the positive invariant region,

$$\begin{aligned} P&= \{ S_{H}, E_{H}, I_{H}, L_A, R_{H}, S_{A},E_{A},I_{A},S_{r},E_{r},I_{r}, L_B,\\&S_{B},E_{B},I_{B},S_{R},E_{R},I_{R} \in \mathbb {R} ^{18}: \\&N_H \le \dfrac{\Pi _H}{\mu _H}, N_v \le \dfrac{\Lambda _1 + \Lambda _2}{\mu _v}\}. \end{aligned}$$

#### Theorem 1

If $N_H(0) > 0$ and $N_v(0) > 0$, the biologically-feasible region P is positively-invariant and all solutions of the model (9) are contained and bounded.

#### Proof

The concepts from [42, 94, 95] are used to provide this proof. First, we define total human and adult mosquito populations are

10 $$\begin{aligned} \dot{N_H}&= \dot{S_H} + \dot{E_H} + \dot{I_H} + \dot{R_H} \end{aligned}$$

11 $$\begin{aligned} \dot{N_1}&= \dot{S_A} + \dot{E_A} + \dot{I_A} + \dot{R_A}+\dot{S_r} + \dot{E_r} + \dot{I_r} \end{aligned}$$

12 $$\begin{aligned} \dot{N_2}&= \dot{S_B} + \dot{E_B} + \dot{I_B} + \dot{R_B}+\dot{S_R} + \dot{E_R} + \dot{I_R} \end{aligned}$$

By substituting the equations (9) into equations 10, 11 and 12, we have

$$\begin{aligned} \dot{N_H}&= \Pi _H -\mu _H (S_H+E_H+I_H+R_H)-\tau I_H, \\&=\Pi _H -\mu _H N_H-\tau I_H \le \Pi _H -\mu _H N_H \le \dfrac{\Pi _H}{\mu _H} \end{aligned}$$

On the other hand,

$$\begin{aligned} \dot{N_v}&= \dot{N_1} + \dot{N_2}, \\ \dot{N_v}&= \Lambda _1 -(\mu _A (S_A+E_A+I_A)+\mu _r(S_r+ E_r+I_r)) \\&+ \Lambda _2 -(\mu _B (S_B+E_B+I_B)+\mu _R(S_R+ E_R+I_R)), \\&= \Lambda _1 + \Lambda _2 -\mu _v(N_1 +N_2) \ \text {where} \ \mu _v=\min (\mu _A, \mu _r, \mu _B, \mu _R), \\ \Rightarrow&N_v \le \dfrac{\Lambda _1+ \Lambda _2}{\mu _v}. \end{aligned}$$

$$\square $$

This implies that, the non-negative solutions of human and mosquito population for the system (9) exist, and are contained in the positively-invariant region P.

### Malaria free equilibrium

The malaria-free equilibrium (MFE) is solved when the left-hand side of the system (9) equals zero. The MFE occurs when neither human nor mosquito populations are infected with the malaria parasite. Thus, it is represented by

$$\begin{aligned} E_{0}&= (S_{H}^0,0,0,0,S_{A}^0,0,0,S_{r}^0,0,0, S_{B}^0,0,0, S_{R}^0,0,0), \ \ \ \ \text {where,} \\ S_{H}^0&= \dfrac{\pi _H}{\mu _H}, \ \ S_{A}^0 = \dfrac{\Lambda _1}{\mu _A}-(\dfrac{\phi \Lambda _1 \mu _{A} \mu _r + \sigma _A \Lambda _1 \mu _r}{\sigma _A \mu _r \mu _A +(\mu _r+\sigma _r) \mu _{A}^2}), \\&S_{r}^0= \dfrac{\phi \Lambda _1 \mu _{A} + \sigma _A \Lambda _1}{\sigma _A \mu _r + (\mu _r+\sigma _r) \mu _{A}}, \\ S_{B}^0&= \dfrac{\Lambda _2}{\mu _B}-(\dfrac{\Phi \Lambda _2 \mu _{B} \mu _R + \sigma _B \Lambda _2 \mu _R}{\sigma _B \mu _R \mu _B +(\mu _R+\sigma _R) \mu _{B}^2}), \\&S_{R}^0= \dfrac{\Phi \Lambda _2 \mu _{B} + \sigma _B \Lambda _2}{\sigma _B \mu _R + (\mu _R+\sigma _R) \mu _{B}}. \end{aligned}$$

### Reproduction numbers

The basic reproduction number ($R_0$) and effective reproduction number ($R_e$) are key metrics in epidemiology [96, 97]. $R_0$ represents the transmission potential in a fully susceptible population without interventions, while $R_e$ reflects actual transmission, taking interventions into account. In this study, we used the next generation matrix approach by P. van den Driessche and Watmough [98] to derive the reproduction numbers. By defining $\mathbb {F}$ and $\mathbb {V}$ as the column matrices of new infections and transfer of infection from one compartment to another, respectively. The reproduction number is the spectral radius of the next generation matrix given by

$$\begin{aligned} FV^{-1}&= \Big [\dfrac{\partial \mathbb {F} (E_{0})}{\partial x_i}\Big ] \Big [\dfrac{\partial \mathbb {V}(E_{0})}{\partial x_i}\Big ]^{-1}, \ \ \text {where} \\&\ \ x_i \in (E_H,I_H, E_A, I_A,E_r, I_r,E_B, I_B, E_R, I_R). \end{aligned}$$

As a result,

$$ F = \begin{pmatrix} 0 &  0 &  0 &  P_0 \beta _A S_H &  0 &  P_0 \beta _r S_H &  0 &  P_0 \beta _B S_H &  0 &  P_0 \beta _R S_H \\ 0 &  0 &  0 &  0 &  0 &  0 &  0 &  0 &  0 &  0 \\ 0 &  P_1 \beta _A S_A &  0 &  0 &  0 &  0 &  0 &  0 &  0 &  0 \\ 0 &  0 &  0 &  0 &  0 &  0 &  0 &  0 &  0 &  0 \\ 0 &  P_1 \beta _r S_r &  0 &  0 &  0 &  0 &  0 &  0 &  0 &  0 \\ 0 &  0 &  0 &  0 &  0 &  0 &  0 &  0 &  0 &  0 \\ 0 &  P_1 \beta _B S_B &  0 &  0 &  0 &  0 &  0 &  0 &  0 &  0 \\ 0 &  0 &  0 &  0 &  0 &  0 &  0 &  0 &  0 &  0 \\ 0 &  P_1 \beta _R S_R &  0 &  0 &  0 &  0 &  0 &  0 &  0 &  0 \\ 0 &  0 &  0 &  0 &  0 &  0 &  0 &  0 &  0 &  0 \end{pmatrix} $$

$$ V = \begin{pmatrix} Q_1 &  0 &  0 &  0 &  0 &  0 &  0 &  0 &  0 &  0 \\ -\epsilon _H &  Q_2 &  0 &  0 &  0 &  0 &  0 &  0 &  0 &  0 \\ 0 &  0 &  Q_3 &  0 &  -\sigma _r &  0 &  0 &  0 &  0 &  0 \\ 0 &  0 &  -\epsilon _1 &  Q_4 &  0 &  -\sigma _r &  0 &  0 &  0 &  0 \\ 0 &  0 &  -\sigma _A &  0 &  Q_5 &  0 &  0 &  0 &  0 &  0 \\ 0 &  0 &  0 &  -\sigma _A &  -\epsilon _2 &  Q_6 &  0 &  0 &  0 &  0 \\ 0 &  0 &  0 &  0 &  0 &  0 &  Q_7 &  0 &  -\sigma _R &  0 \\ 0 &  0 &  0 &  0 &  0 &  0 &  -\epsilon _3 &  Q_8 &  0 &  -\sigma _R \\ 0 &  0 &  0 &  0 &  0 &  0 &  -\sigma _B &  0 &  Q_9 &  0 \\ 0 &  0 &  0 &  0 &  0 &  0 &  0 &  -\sigma _B &  0 &  Q_{10} \end{pmatrix} $$

Thus, the effetive reproduction number $R_e$ is

13 $$\begin{aligned} \rho (FV^{-1})= \sqrt{L_1 L_{9}+ L_3 L_{11} + L_5 L_{13}+ L_7 L_{15}}. \end{aligned}$$

Where,

$$\begin{aligned} Q_1&=\epsilon _H + \mu _H, \ \ Q_2 =\tau + \gamma + \mu _H, \ \ \\&Q_3 =\epsilon _1+\mu _{A} + \sigma _{A} , \ \ Q_4= \mu _{A} + \sigma _{A}, \\ Q_5&= \epsilon _2 + \mu _r + \sigma _r, \ \ Q_6 = \mu _r + \sigma _r, \ \ Q_7= \mu _{B} + \epsilon _2 + \sigma _B, \\ Q_8&= \sigma _B + \mu _B, \ \ Q_9= \mu _R + \epsilon _4 + \sigma _R, \ \ \ Q_{10}=\sigma _R + \mu _R,\\ L_{1}&= \dfrac{P_0 \beta _A \pi _H (Q_5 Q_6 \epsilon _1+\epsilon _2 \sigma _A \sigma _r)}{\mu _H(Q_3 Q_5-\sigma _A \sigma _r) (Q_4 Q_6-\sigma _A \sigma _r)} \\&+\dfrac{P_0 \beta _r \pi _H \sigma _A (Q_4 \epsilon _2+Q_5\epsilon _1)}{\mu _H(Q_3 Q_5-\sigma _A \sigma _r)(Q_4 Q_6-\sigma _A \sigma _r)},\\\\ L_2&= \dfrac{P_0 \beta _A \pi _H Q_6}{\mu _H(Q_4 Q_6-\sigma _A \sigma _r)}+ \dfrac{P_0 \beta _r \pi _H \sigma _A }{\mu _H(Q_4 Q_6-\sigma _A \sigma _r)}, \ \\&L_4= \dfrac{P_0 \beta _A \pi _H \sigma _r}{\mu _H(Q_4 Q_6-\sigma _A \sigma _r)} +\dfrac{P_0 \beta _r \pi _H Q_4}{\mu _H(Q_4 Q_6-\sigma _A \sigma _r)}, \\\\ \end{aligned}$$

$$\begin{aligned} L_3&= \dfrac{P_0 \beta _A \pi _H \sigma _r (Q_3 \epsilon _2+Q_6 \epsilon _1)}{\mu _H(Q_3 Q_5-\sigma _A \sigma _r) (Q_4 Q_6-\sigma _A \sigma _r)} \\&+ \dfrac{P_0 \beta _r \pi _H(Q_3 Q_4 \epsilon _2+\epsilon _1 \sigma _A \sigma _r) }{\mu _H(Q_3 Q_5-\sigma _A \sigma _r)(Q_4 Q_6-\sigma _A \sigma _r)}, \\\\ L_5&= \dfrac{P_0 \beta _B \pi _H \epsilon _3 Q_9 Q_{10}}{\mu _H(Q_7 Q_9-\sigma _B \sigma _R)(Q_8 Q_{10}-\sigma _B \sigma _R)}\\&+\dfrac{P_0 \beta _R \pi _H \sigma _B \epsilon _3 Q_9}{\mu _H(Q_7 Q_9-\sigma _B \sigma _R)(Q_8 Q_{10}-\sigma _B \sigma _R)}, \\\\ L_6&= \dfrac{P_0 \beta _B \pi _H Q_{10}}{\mu _H(Q_8 Q_{10}-\sigma _B \sigma _R)} + \dfrac{P_0 \beta _R \pi _H \sigma _B}{\mu _H(Q_8 Q_{10}-\sigma _B \sigma _R)}, \\&L_8= \dfrac{P_0 \beta _B \pi _H \sigma _R}{\mu _H (Q_8 Q_{10}-\sigma _B \sigma _R)} +\dfrac{P_0 \beta _R \pi _H Q_8}{\mu _H (Q_8 Q_{10}-\sigma _B \sigma _R)}, \\\\ L_7&= \dfrac{P_0 \beta _B \pi _H \epsilon _3 \sigma _R Q_{10}}{\mu _H(Q_7 Q_9-\sigma _B \sigma _R)(Q_8 Q_{10}-\sigma _B \sigma _R)}\\&+ \dfrac{P_0 \beta _R \pi _H \sigma _B \epsilon _3 \sigma _R}{\mu _H (Q_7 Q_9-\sigma _B \sigma _R)(Q_8 Q_{10}-\sigma _B \sigma _R)}, \\\\ L_9&= \dfrac{P_1 \beta _A \epsilon _H}{Q_1 Q_{2}}\Big (\dfrac{\Lambda _1}{\mu _A}-(\dfrac{\phi \Lambda _1 \mu _{A} \mu _r + \sigma _A \Lambda _1 \mu _r}{\sigma _A \mu _r \mu _A +(\mu _r+\sigma _r) \mu _{A}^2})\Big ), \\&L_{10} = \dfrac{P_1 \beta _A}{Q_{2}} \Big (\dfrac{\Lambda _1}{\mu _A}-(\dfrac{\phi \Lambda _1 \mu _{A} \mu _r + \sigma _A \Lambda _1 \mu _r}{\sigma _A \mu _r \mu _A +(\mu _r+\sigma _r) \mu _{A}^2})\Big ), \\\\ L_{11}&= \dfrac{P_1 \beta _r \epsilon _H}{Q_1 Q_{2}} \Big (\dfrac{\phi \Lambda _1 \mu _{A} + \sigma _A \Lambda _1}{\sigma _A \mu _r + (\mu _r+\sigma _r) \mu _{A}} \Big ), \\&L_{12} = \dfrac{P_1 \beta _r}{Q_{2}} \Big (\dfrac{\phi \Lambda _1 \mu _{A} + \sigma _A \Lambda _1}{\sigma _A \mu _r + (\mu _r+\sigma _r) \mu _{A}} \Big ), \\\\ L_{13}&= \dfrac{P_1 \beta _B \epsilon _H}{Q_1 Q_{2}} \Big ( \dfrac{\Lambda _2}{\mu _B}-(\dfrac{\Phi \Lambda _2 \mu _{B} \mu _R + \sigma _B \Lambda _2 \mu _R}{\sigma _B \mu _R \mu _B +(\mu _R+\sigma _R) \mu _{B}^2})\Big ), \\&L_{14} = \dfrac{P_1 \beta _B }{Q_{2}} \Big ( \dfrac{\Lambda _2}{\mu _B}-(\dfrac{\Phi \Lambda _2 \mu _{B} \mu _R + \sigma _B \Lambda _2 \mu _R}{\sigma _B \mu _R \mu _B +(\mu _R+\sigma _R) \mu _{B}^2})\Big ), \\\\ L_{15}&= \dfrac{P_1 \beta _R \epsilon _H}{Q_1 Q_{2}} \Big (\dfrac{\Phi \Lambda _2 \mu _{B} + \sigma _B \Lambda _2}{\sigma _B \mu _R + (\mu _R+\sigma _R) \mu _{B}}\Big ), \\&L_{16} = \dfrac{P_1 \beta _R }{Q_{2}} \Big (\dfrac{\Phi \Lambda _2 \mu _{B} + \sigma _B \Lambda _2}{\sigma _B \mu _R + (\mu _R+\sigma _R) \mu _{B}}\Big ). \end{aligned}$$

Effective reproduction number $(R_e)$ indicates the average number of new cases of malaria produced by potentially infectious individuals when the interventions are in operation. When no interventions utilisation or coverage, the $R_e$ becomes basic reproduction number ($R_0$).

### Local and global stability of malaria free equlibrium

#### Theorem 2

If $R_e < 1$, the MFE defined by $E_0$ is locally asymptotically stable in the region defined by system (9), it is unstable if $R_e > 1$.

#### Theorem 3

The MFE defined by $E_0$ is globally asymptotically stable in the region defined by system (9) if $R_e < 1$, it is unstable if $R_e > 1$.

#### Proof

We provide the proof using the comparison theorems for differential equations in [99, 100]. The infected variables from system (9) can be expressed as matrices A and B as follows:

A= $$({\dot{E}_{H}} \ \ {\dot{I}_{H}} \ \ {\dot{E}_{A}} \ \ {\dot{I}_{A}} \ \ {\dot{E}_{r}} \ {\dot{I}_{r}} \ \ {\dot{E}_{B}} \ {\dot{I}_{B}} \ \ {\dot{E}_{R}} \ \ {\dot{I}_{R}})^T$$, B= $$(E_{H} \ \ I_{H} \ \ E_{A} \ \ I_{A} \ \ E_{r} \ I_{r} \ \ E_{B} \ I_{B} \ \ E_{R} \ \ I_{R})^T$$. Then,

$$\begin{aligned} A= (F-V) B- F B \Rightarrow B \le (F-V) B, \end{aligned}$$

where F and V are Jacobian matrices 1. All eigenvalues of the matrix (F-V) have negative real parts, following the local stability of MFE, the system (9) is stable whenever $R_e<1$. Thus, as time (t)$\longrightarrow$ $\infty$, then

$(S_{H}, E_{H}, I_{H}, R_{H}, S_{A},E_{A},I_{A},S_{r},E_{r},I_{r}, S_{B},E_{B},I_{B},S_{R},E_{R},I_{R}) \longrightarrow$ $E_0$ (for more details, see comparison theorems in [99, 100]).

Therefore, $E_0$ is globally asymptotically stable whenever $R_e<1$. $\square$
